# The Impact of Climate-Change-Related Disasters on Africa’s Economic Growth, Agriculture, and Conflicts: Can Humanitarian Aid and Food Assistance Offset the Damage?

**DOI:** 10.3390/ijerph19010467

**Published:** 2022-01-01

**Authors:** Go Shimada

**Affiliations:** School of Information and Communication, Meiji University, Chiyoda-ku, Tokyo 101-8301, Japan; go_shimada@meiji.ac.jp

**Keywords:** climate change, natural disasters, agricultural production, food aid, official development assistance, conflict, poverty, cereal production, humanitarian aid

## Abstract

This study analyzed the impact of climate-related natural disasters (droughts, floods, storms/rainstorms) on economic and social variables. As the Africa-specific empirical literature is limited, this study used panel data from 1961–2011 on Africa. The study used a panel data regression model analysis. The results showed that climate change-related natural disasters affected Africa’s economic growth, agriculture, and poverty and caused armed conflicts. Among the disasters, droughts are the main cause of negative impact, severely affecting crops such as maize and coffee and resulting in increased urban poverty and armed conflicts. In contrast, international aid has a positive effect but the impact is insignificant compared to the negative consequences of climate-related natural disasters. Cereal food assistance has a negative crowding-out effect on cereal production. International donors should review their interventions to support Africa’s adaptative capacity to disasters. Government efficiency has reduced the number of deaths, and this is an area that supports Africa’s adaptative efforts.

## 1. Introduction

With the increasing threat of global warming, there is a drastic increase in the number of catastrophic natural disasters. Every year, irregular and extreme weather events are reported worldwide. In 2021, Europe experienced an oppressive heatwave and was hit by devastating floods in July [1]. The magnitude of destruction was severe in Germany and Belgium, causing several deaths. In Asia, tropical cyclones have become stronger in recent years [2]. Additionally, Japan has recorded torrential rainfall, flooding, and landslides more often than before. Global warming is a worldwide phenomenon, but African countries are disproportionately punished, even though they contributed the least toward greenhouse gas emissions compared with developed countries. Therefore, do donor countries contribute sufficient aid to African countries to promote Africa’s adaptive capacity? To answer this question, we must understand the extent of the collateral damage of global warming in Africa. This study analyzed the damage caused by climate-related natural disasters, such as floods, droughts, and storms. (The phenomenon of extreme temperature is not included in this study, as the frequency is shallow in Africa.) The remainder of this paper is organized as follows. Section 1 examines the current damage trends caused by climate-related natural disasters. Section 2 presents the study methods and data. Section 3 discusses the analysis results. Based on the results, the discussion is presented in Section 4. Finally, conclusions are drawn from the results and discussion.

### 1.1. Background: Climate Change and Natural Disasters in Africa

This section reviews climate change and natural disasters in Africa. Subsequently, it reviews the existing literature on the impact of climate change and related natural disasters [3,4]. For instance, using satellite images, Tellman et al. [5] found that the majority of the population was exposed to floods between 2000 and 2015, especially in Asia and sub-Saharan Africa. The authors also argued that their projections for 2030 indicate that a larger number of people will be exposed to flood threats.

### 1.2. GDP Per Capita

Gross domestic product (GDP) per capita helps measure the economic and social impact of climate-related natural disasters in Africa. There are several studies on the overall impact of natural disasters on economic growth, but not specific to climate-related disasters [6,7,8,9,10]. While there is no consensus regarding its impact on economic growth, some found a significantly negative long-term effect [11,12,13,14,15,16]. Conversely, some studies show a positive impact on economic growth as disasters promote the “Schumpeterian creative destruction” process [17,18,19,20,21]. According to this view, disasters promote innovation and investment, destroying existing practices, products, or services. Other studies reveal mixed results on economic growth due to natural disasters [22,23]. Therefore, it is necessary to clarify the effect of climate-related disasters in Africa.

### 1.3. Agricultural Production and Aid

The socioeconomic impact on agriculture and food security have been identified as critical sectors in the age of climate change [24]. Lesk et al. [25] found that droughts have reduced national cereal production by 9–10% in terms of impact on crop production. However, they found statistically no significant effect of floods. Nonetheless, the question arises if this is truly a global phenomenon or specific to Africa.

Africa is particularly vulnerable to climate change, as the region’s adaptive capacity has certain constraints [26,27,28,29,30]. Notably, most farmers in Africa are small-holder farmers without adequate education or skills to adapt to warming temperatures and damages caused by natural disasters [31]. As there is limited empirical researched, this study examines the impact of climate-related disasters on African agriculture because this sector is most critical for people’s livelihoods compared to other sectors. Therefore, if disasters decrease agricultural production, it increases poverty in both rural and urban areas.

In this regard, the literature does not consider social variables. Agricultural production is not determined by disasters. Other factors, such as farmers’ human capital and market demand for agricultural commodities, are essential production factors [32,33]. The prior literature does not control for these social variables and may produce biased results. Therefore, this study estimated the impact of climate-related disasters by controlling for these factors.

A critical control variable is disaster relief provided by international donors, including the United Nations (UN), World Bank, and bilateral governments. Some literature is available on disaster relief based on case studies [34]. In contrast, there are few quantitative studies, especially on how disaster relief mitigates damage or contributes toward recovery [35,36]. One study [35] found that increased foreign aid resulted in higher fatality rates. According to another study [36], international aid increases social strife rather than decreasing it, promoting a new conflict over the distribution of resources.

Some studies have shown that aid increased after a disaster [7,37]. (There are some studies on the impact of domestic government aid to mitigate the damage, but not international aid (see, for example, [38]).) However, none applied an aid disaggregation approach to examine the impact of different aid types. These studies only used aggregated aid data. This is challenging because aid has a distinct impact. For instance, aid for primary education shows different results from assistance provided for infrastructure or humanitarian food aid. If this study uses aggregated aid data, it may lead to erroneous policy recommendations. For instance, assistance for infrastructural growth tends to expand in budgets compared to agricultural development aid. It is essential to distinguish between various aid types. Therefore, there is a research gap in this regard. There is no study on whether humanitarian aid, food aid, and other assistance forms have mitigated the damage caused by natural disasters.

### 1.4. Impact on Poverty and Armed Conflict

Regarding the impact on poverty, Kahn [6] found that the Gini coefficient is positively correlated with disaster-related deaths. As Barrett [39] discussed, food insecurity is associated with sudden catastrophe-like disasters and chronic poverty. Damage due to disasters is of two types: direct and indirect. Notably, in direct damage, the disaster itself kills people. In indirect damage, there are cases where people die not because of the disaster itself but due to displacement (i.e., losing their jobs after the disaster) and resultant poverty. Therefore, it is essential to measure the impact of such indirect consequences.

There is no consensus in the previous literature on the natural disaster–conflict nexus. Some studies found a link, whereas some others did not. For instance, O’Loughlin et al. [40] studied the link between climate variability and armed conflicts and found that, in general, extremely high temperatures are associated with greater conflict levels. They also found that the link varies depending on the conflict type and different subregions of Africa. Burke et al. [41] found a strong historical association between civil war and temperature in Africa, indicating that by 2030 armed conflict is likely to increase by approximately 54%. However, Buhaug [42] argued against this nexus, reporting that the incidence of armed conflict has declined in Africa since 2002 despite rising temperature levels. Following this counterargument, Burke et al. [43] stated that there are some econometric issues in Buhaug’s [42] study. (Burke also admits that the climate–conflict nexus still stands, but the nexus has weakened since 2002.)

These previous studies examined the temperature–conflict nexus. However, this nexus does not have a direct causal relationship. There are several indirect links between the two: natural disasters and vegetation. High temperatures cause crop damage, and damage occurs when the temperature is above 30 °C or when the average temperature is above 25 °C for a prolonged period. Tolerance to high temperatures varies among crops. Therefore, these two consequences would have some social impact, such as reducing income. Consequently, such outcomes potentially lead to conflicts. The past literature did not consider the indirect causal relationship and treats the temperature–conflict link as a black box. The impact of high temperatures on vegetation was beyond the scope of this study; therefore, this research examined the impact of disasters on conflicts.

### 1.5. Government Effectiveness

Strömberg [7] studied whether government effectiveness is essential for dealing with disasters. The study used the government effectiveness index, an indicator produced by the World Bank, to test its importance of governance effectiveness. Government effectiveness is essential during disasters. At the time of a crisis, the government needed to handle everything quickly and within a limited period. Even in developed countries, handling crises after disasters is challenging and sometimes governments fail to cope with them. However, this is likely more difficult for developing countries. This raises the question: How important is government effectiveness in Africa? Strömberg found that government effectiveness reduces the number of people killed by natural disasters globally and not specifically in Africa. This study examined the importance of government effectiveness to mitigate damage caused by climate-related disasters.

This section reviewed the current trends in climate-related natural disasters in Africa and examined the literature on the impact of disasters on GDP, agriculture, poverty and conflict, and government effectiveness.

First, based on the identified research gaps, the next section examines the impact of climate-related natural disasters and international aid on GDP per capita. Second, the nexus between disaster–agriculture is discussed, focusing on major crops. Third, the impact on poverty and conflict was tested. Finally, factors contributing to decreasing (or increasing) the death toll due to natural disasters were tested. One of the factors examined was international aid extended to African countries.

Based on the research gap identified in this section, the next section discusses the following. First, it overviews the impact of climate-related natural disasters and international aid on GDP per capita. Second, the nexus between the disasters–agriculture is discussed, focusing on major crops. Third, the impacts on poverty and conflicts are investigated. Finally, factors contributing to decreasing (or increasing) death tolls by natural disasters are tested. One of the factors examined is international aid extended to African countries.

## 2. Methods and Data

This section describes the analytical framework for empirical analysis. This study tested the impact of three elements of climate-related disasters on (1) GDP per capita and agricultural production, (2) impact on poverty and conflict, and (3) factors contributing toward reducing the impact of disasters.

Research gaps were identified based on the literature review in Section 1. There is no consensus on the impact of natural disasters on GDP, and there is no empirical analysis focusing on the impact of climate-related natural disasters on agricultural production in Africa. Even the literature on global agricultural production does not control for other socio-economic conditions. Agricultural production is a part of economic activities; therefore, there is a need to control for these variables. Otherwise, there is a potential for result bias. Therefore, it is important to understand the impact of climate-related natural disasters on GDP and agricultural production, considering other socio-economic factors.

To estimate the impact on GDP per capita growth, the following formula was used, following the model used by Skidmore and Toya [20].

(1) $$\Delta {\left(\frac{Y}{P}\right)}_{i,t}=\alpha_{i,t}+\beta_{1}\Delta {\left(\frac{Y}{P}\right)}_{i,\;t-1}+\beta_{2}Dis_{i,t}+\beta_{3}Aid_{i,t}+\beta_{4}Gov_{i,t}+\beta_{5}X_{i,t}+\varepsilon_{i,t}$$

*Y* denotes GDP and *P* represents the population. Therefore, $\Delta {\left(\frac{Y}{P}\right)}_{i,t}$ is GDP per capita growth, *i* is a country index to capture country-specific effects, and *t* is the time (year) index. The lagged GDP per capita growth ($\Delta \left(\frac{Y}{P}{)}_{i,\;t-1}\right)$ is included because the previous year’s growth trend greatly affects the current year’s economic activities. *Dis_i,t_* is a measure of the impact of disasters specific to country *i* at time *t*. This study used the number of people affected by disasters for this variable. This is because these data represent the impact of disasters. The number of occurrences does not necessarily equate to the impact. If a disaster occurs in an uninhabited area, then the impact on human activities is limited, as people do not live there. *Aid* denotes international aid. This is global official development assistance (ODA) data and not a specific country’s ODA. This study used a different type of aid data, as impact varies depending on the aid type. For instance, the impact of cereal food aid is not the same as a medical aid. Therefore, it is necessary to disaggregate aid data. This study used the following data: aid for agriculture, humanitarian aid, and cereal food aid. *Gov_i,t_* denotes government expenditure. *X_i,t_* are the other control variables, including the following variables: education, fertility rate, and government effectiveness index. The education variable represents human capital [32,33,34,35,36,37,38,39,40,41,42,43,44]. This study used the government effectiveness index developed by the World Bank, which measures the quality of public services, infrastructure, and civil service based on the World Bank’s survey [45].

The following analytical framework will be used to estimate the impacts on agricultural production, reformulating Equation (1) to focus on agriculture.

(2) $$Agr_{i,t}=\alpha_{i,t}+\beta_{1}Dis_{i,t}+\beta_{2}\Delta {\left(\frac{Y}{P}\right)}_{i,t}+\beta_{3}Aid_{i,t}+\beta_{4}X_{i,t}+\varepsilon_{i,t}$$

*Agr_i,t_* denotes the variable for agricultural production. *Dis_i,t_*, $\Delta {\left(\frac{Y}{P}\right)}_{i,t}$, and *Aid_i,t_* indicate the same as the above equation. $\Delta {\left(\frac{Y}{P}\right)}_{i,t}$ is included because agricultural production is affected by economic activities. *X_i,t_* denotes other control variables, such as inequality in educational attainment and the government effectiveness variable. The former represents human capital.

As this estimation used panel data, three methods were used to assess the results. (This study used Stata/SE 17.0 for the estimation.) They estimate fixed effects (FE), random effects (RE), and pooling. Among the three estimation methods, the most appropriate estimation method is determined by the results of the F-test, Hausman test, and the Breusch–Pagan test. The method determined for each model is reported at the bottom of the result tables.

Table 1 shows the descriptive statistics of the data used in the empirical study. This dataset covers 90 African countries (including both sub-Saharan African and North African countries). This is unbalanced panel data. The period varies depending on the data, and they are annual data with gaps. This dataset was constructed using the following four datasets.

First, the Emergency Events Database (EM-DAT) was used for natural disaster-related data [46]. The EM-DAT is an international disasters’ database widely used to analyze natural disasters and has been managed by the Center for Research on the Epidemiology of Disasters (CRED) since 1988. For disasters to be recorded as an extreme event, one of the following criteria must be met: (1) 10 or more people reportedly killed, (2) 100 or more people affected, (3) a declaration of a state of emergency, and (4) a call for international assistance.

Second, the WDI (World Development Indicators) dataset is compiled by the World Bank [45]. The Food and Agriculture Organization Corporate Statistical Database (FAOSTAT) is a dataset on agricultural production provided by the Food and Agriculture Organization (FAO) [47]. Finally, the V-dem is used, produced by the Varieties of Democracy Project on government effectiveness and educational inequality data [48].

Five types of variables are used as measures for disaster impact. The four variables represent the number of people affected by the following: (1) climate-related disasters (aggregate variable), (2) droughts, (3) floods, and (4) storms. The variable of climate-related disasters is the aggregate variable of droughts, floods, and storms. This study also used the number of deaths caused by climate-related disasters. In addition, this study used two other variables: corruption control and the local government index as measures of government effectiveness. If corruption control is inadequate, the government’s effectiveness is considered low and would affect the implementation of recovery after a disaster. For international aid, seven different types of data were prepared to examine how aid works to mitigate the impact of natural disasters. The aid for agricultural development works differently from humanitarian aid. For agricultural production, the agricultural production index is the aggregate index. Production data for major cereals, such as maize, sorghum, and millet, were used to examine the impact of crops.

## 3. Results

**Figure 1:**
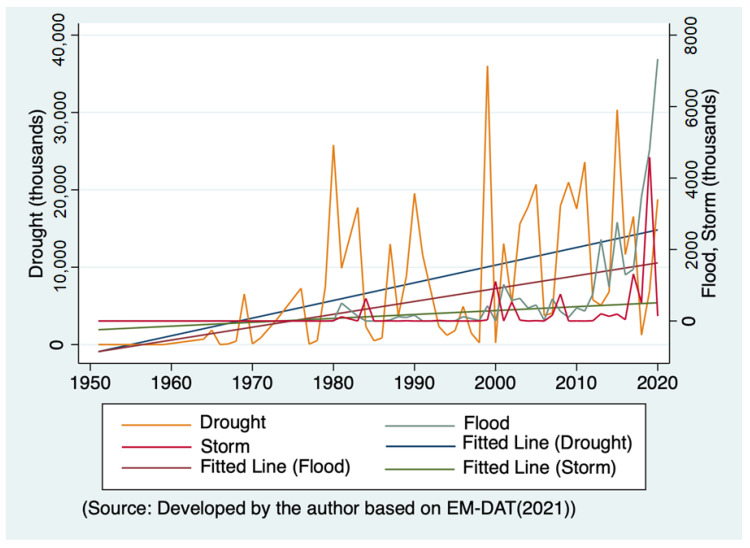
The total number of people affected by droughts, floods, and storms in Africa.

This section reviews the current trends of natural disasters in Africa before analyzing the impacts of natural disasters. Figure 1 shows the number of people affected by droughts, floods, and storms caused by climate change. The data used the Emergency Events Database (EM-DAT), mentioned in the previous section [4]. As a measure of natural disasters, Figure 1 uses the number of people affected rather than the number of disasters that occurred because this is a better measure of disaster severity. Each disaster is different in scale and impact. If a disaster occurs in a remote mountainous area, the social impact is less, but the same type of event would have a devastating effect in an urban area. Therefore, Figure 1 shows the number of people affected, rather than the number of occurrences.

Furthermore, an essential point must be considered when interpreting these numbers. The disaster number, such as the number of people affected by a disaster, may be underreported compared with the actual situation [7]. For instance, some authoritarian African regimes may underreport the damage caused by disasters to avoid being criticized for their response. Given African governments’ capacity for data authenticity, there may be misreporting across countries and over time [49]. In such a case, there is an underlying risk of underestimating the significance of the impact of climate-related disasters.

Figure 1 has two axes because the damage caused by droughts is much larger than that caused by floods and storms. The left axis represents the total number of people affected by droughts, and the right axis shows the number of people displaced by floods and storms. The worst drought affected more than 35,000 people in 1999. However, on the other hand, the impact of floods and storms is much lower than the drought. Due to the nature of disasters, all three lines, representing each natural disaster, fluctuate significantly. For instance, there was a massive drought in 1999, but there was no drought before and after the year. The damage caused by droughts was not significant until the late 1960s, and they seem to occur quite often now. A similar pattern can be observed for floods and storms. However, during the late 1990s, there was an unprecedented increase in the number of people affected by floods and storms.

To clearly understand the long-term trend of natural disasters, fitted lines for drought, floods, and storms are drawn in Figure 1. These three fitted lines show a clear upward trend, especially due to droughts, followed by floods and storms. Therefore, as Figure 1 confirms, the damage caused by climate-related disasters has increased rapidly since the late 1960s, primarily during droughts. Therefore, the next question is how disaster-related damage affects Africa’s socio-economic development and growth.

Table 2 presents the estimated coefficients in the impact on GDP per capita growth, and the difference among the four models is the difference of data used. Models 1 and 2 used aggregate disaster measures (number of people affected by climate-related disasters) and Models 3 and 4 used disaggregated disaster variables (droughts, floods, and storms). As the dataset is unbalanced panel data with gaps, the *N* used for estimation differs depending on the variable used. Models 1 and 2 show that climate-related natural disasters significantly lowered GDP per capita growth. However, as Models 3 and 4 show, the impact differs depending on the natural disaster type. Only droughts had a statistically significant negative impact, as opposed to floods and storms. This indicates the importance of any drought policy to sustain GDP per capita growth in Africa.

Models 1 and 3 examined the impact of humanitarian ODA on GDP per capita. How do global efforts mitigate the negative impact of natural disasters? Similar to Models 2 and 4, which examined the emergency aid, all these variables became insignificant. This is reasonable considering that, compared with the size of a country’s GDP, the amount of aid in these categories is too small to capture the impact. Therefore, the study next examined aid impact focusing on crops because the agricultural sector is particularly vulnerable to natural disasters, affecting economic growth and damaging crop production.

Table 3 examines the impact on agricultural production. The number of people affected by natural disasters, an aggregate variable, is used to measure disaster impact. This variable is statistically significant, and the negative coefficient is also substantial. This result indicates that climate-related disasters have a significant negative impact on agricultural production. Educational inequality is also negative. Therefore, human capital is important for agricultural production. As the educational Gini coefficient reflects income inequality, the widening rich–poor gap negatively impacts agricultural production. This is consistent with past studies [32,33]. Therefore, human capital is required to cope with disasters caused by climate change, which are likely to increase in the future.

According to Models 1 and 4, humanitarian aid and ODA for disaster prevention and preparedness are not statistically significant. In contrast, according to Models 2 and 3, agricultural aid is positive. These results are consistent with the expected outcome, as humanitarian aid and disaster prevention assistance do not support agricultural production. However, this analysis shows that disasters have a substantial negative impact on agricultural production. Therefore, agricultural aid has become even more important during the age of global warming, specifically to fight against the long-term negative consequences. However, it is necessary to note that the coefficient of agricultural aid is small, indicating that agricultural aid alone is not enough to mitigate the negative impact of climate-related disasters.

Table 4 investigates the impact of climate-related disasters on agriculture. Compared to Table 3, the impact of different types of aid is tested in Table 4. Models 1, 2, and 3 examine emergency aid, agricultural aid, and cereal food aid, respectively. Emergency aid is not significant, similar to humanitarian aid. This is expected because emergency aid does not aim at agricultural development. The impact on agricultural aid and cereal food aid is in the opposite direction, wherein agricultural aid is positive but cereal food aid is negative due to a substitutional effect. The inflow of cereals from foreign countries seems to have a crowding-out effect on domestically produced cereals. For agricultural aid, even if the coefficient is positive, it is very small and unremarkable compared to the coefficient of disasters. In other words, agricultural aid does not compensate for the negative impact of disasters. The results for the impact of disasters and educational inequality are the same as those in Table 3, further confirming the earlier results.

Table 5 and Table 6 examine the impact on a crop-by-crop basis. The difference between these two tables is that Table 5 includes ODA for agriculture, whereas Table 6 examines the impact of cereal aid. This analysis shows that the impact varies by crop. For instance, droughts negatively impact maize. Storms reduce rice and fonio production because, at the heading stage, strong winds can topple the panicles of rice and fonio. Floods also reduce fonio production. However, this result is not robust, as these crops are statistically insignificant (Table 6). Therefore, the impact of disasters on crops varies depending on the vegetation type.

Agricultural ODA has a positive impact on maize, sorghum, millet, and rice, but the impact differs by crop. Furthermore, the coefficient is very small. More importantly, it can be observed in Table 6 that the impact of cereal aid is different from agricultural ODA. Overall, cereal aid has a negative impact, but this influence differs depending on the crop. Maize, sorghum, rice, and wheat are all negatively affected, but millet production increases marginally. However, the reason for increased millet production is unclear, but millet may be used as a substitute for cereals such as wheat, whose production decreased. In many cases, cereal aid is provided during humanitarian crises, including natural disasters. However, cereal aid can negatively affect production because cereals provided through food aid have a crowding-out effect on domestic production.

This study also examined non-cereal crops, such as bananas, cassava, tea, and coffee (Appendix A). It can be observed that only coffee is negatively and strongly affected by droughts because water stress affects coffee production due to water availability sensitivity.

As discussed above, natural disasters negatively impact agricultural production. The next question is what are the consequences of such effects. Table 7 examines the impact of climate-related disasters on poverty and conflicts because reduced agricultural production indicates lower income for farmers. This would probably impact poverty and conflicts. As observed in Section 1 (Literature review), previous studies examine the temperature–conflict link without considering the internal mechanism. As discussed, using disaster data, this study examined the internal nexus between climate change and armed conflicts.

Model 1 examined the impact of aggregate climate-related natural disasters in rural areas and shows that poverty does not increase in rural areas. The growth in GDP per capita reduces poverty in rural areas. However, this situation contrasts in urban areas, where climate-related natural disasters increase poverty. This is probably because people migrate from rural to urban areas in search of jobs after natural disasters occur. Therefore, rather than rural areas, poverty increases in urban areas. Model 3 analyzes the impact of different disaster types, focusing only on urban areas. The results show that climate variability that leads to extreme events such as droughts causes a substantial increase in poverty in Africa.

Amid armed conflicts, another possible outcome is reduced agricultural production. Models 4 and 5 studied the impact of natural disasters on the number of battle-related deaths. Using aggregate natural disaster data, Model 4 shows that while growth in GDP per capita reduced battle-related deaths, natural disasters significantly increased the death toll. Model 5 also confirmed this aspect. Among the types of natural disasters, droughts exacerbated battle-related deaths in Africa.

As these results indicate, natural disasters reduce agricultural production and trigger poverty and armed conflicts.

Finally, Table 8 examines the various factors that contributed toward reducing the number of deaths caused by climate-related natural disasters. This analysis aimed to explore how African countries can respond and mitigate the adverse effects of climate warming.

Model 1 examined HDI (Human Development Index), government effectiveness, and corruption control. Corruption control is a proxy for government transparency. Government effectiveness is strongly significant in reducing the number of disaster-related deaths. The ODA for disaster prevention and preparedness has also become positive. However, the coefficient is not necessarily large enough to mitigate the impact on deaths. Therefore, the government’s capacity building is critical for mitigating the impact of natural disasters. Model 2 includes the regulatory quality of government policies, but the results do not change.

Model 4 tests humanitarian aid, and Model 5 includes emergency aid. Both are statistically significant in reducing the number of deaths caused by natural disasters. This is an excellent outcome for international donors, but it is necessary to note that the coefficients are very small. In other words, to mitigate damage caused by increasing climate-related disasters, these aids are inadequate for coping with the damage. From this analysis, it can be concluded that government effectiveness is key to reducing the number of deaths caused by natural disasters.

## 4. Conclusions

Unlike previous studies, this study controlled for social variables and examined the crop-by-crop impact. Using panel data from African countries, this study found the following four aspects.

1. Climate-related natural disasters negatively impact per capita GDP growth and agricultural production. The impact is severe on cereal production, especially droughts (maize) and storms (rice and fonio).
2. While ODA for agriculture has a slightly positive impact, cereal aid food negatively impacts cereal production (maize, sorghum, rice, wheat).
3. Climate-related disasters, primarily droughts, increase poverty in urban areas and increase battle-related deaths. This result supports Burke et al.’s [41] argument.
4. Finally, government effectiveness is key to determining the number of deaths caused by climate-related disasters.

There are several important policy implications. First, these findings show that climate change has severe consequences not only for the development of African countries but also armed conflicts. As mentioned earlier, Africa is the least responsible for the increase in greenhouse emissions. Therefore, donor countries must initiate quick action to assist African countries and help them cope with climate change, especially climate-related natural disasters. Among natural disasters that are closely associated with human life, droughts have severe consequences on the following socio-economic activities: GDP per capita growth, agricultural production, poverty, and armed conflicts. International donors must focus on developing measures to prevent droughts and assist African countries’ adaptive strategies to combat global warming.

Second, government effectiveness is key to coping with climate-related disasters. International aid must be provided to improve effectiveness. This is clear, as the coefficient of the impact to reduce the number of deaths was small on ODA for disaster prevention and preparedness, humanitarian ODA, and emergency ODA.

Third, there is a need to review if cereal aid is beneficial for African countries as it reduces cereal production, possibly due to a crowding-out effect on domestic production.

As discussed in Section 2, disaster data may be underreported. Most authoritarian governments do not overemphasize the damage caused by natural disasters. These governments have a strong incentive to underreport damages. Thus, our assessment likely underestimates the true damage level. Therefore, it is necessary to plan policies and measures that consider this possibility.

## Figures and Tables

**Table 1:** Descriptive Statistics.

Variable	Obs.	Mean	Std. Dev.	Min.	Max.	Year	Data Source
Number of People Affected by Climate-related Disasters	3394	171,620.9	906,675.7	0	23,000,000	1900–2021	EM-DAT
Number of People Affected by Drought	3143	0.0140703	0.080764	0	1	1900–2021	EM-DAT
Number of People Affected by Flood	3143	0.0024037	0.0179437	0	0	1927–2021	EM-DAT
Number of People Affected by Storm	3143	0.0012416	0.0250523	0	1	1948–2021	EM-DAT
Number of Deaths by Climate-related Disasters	3394	267.0533	6475.562	0	300,000	1900–2021	EM-DAT
Control of Corruption	1185	−0.542972	0.687297	−1.869	2	1996–2019	V-Dem
Local Government Index	3224	0.4376833	0.3243581	0	1	1900–2020	V-Dem
Educational Inequality, Gini	2283	60.83778	22.18283	11.875	99.804	1927–2010	V-Dem
Net ODA	2286	10.48105	11.82156	−0.251879	147	1960–2011	WDI
Humanitarian ODA	503	66,800,000	171,000,000	1387	1,380,000,000	2002–2011	WDI
ODA for reconstruction relief and rehabilitation	296	5,796,040	13,200,000	−52,185	96,900,000	2002–2011	WDI
ODA for Agriculture	513	29,000,000	40,400,000	7069	387,000,000	2002–2011	WDI
Emergency ODA	493	64,100,000	165,000,000	1387	1,280,000,000	2002–2011	WDI
ODA for Disaster Prevention and Preparedness	249	1,209,090	2,266,356	−75,420	16,700,000	2002–2011	WDI
Cereal Food Aid	1309	67,786.64	165,895	0	1,900,805	1988–2012	WDI
Agriculture Production Index	2583	70.33316	28.66801	13.42	193	1961–2011	WDI
Cereal Production Index	2512	82.68087	77.53881	5.79	1925	1961–2011	WDI
Maize Production (ton)	1950	584,838.4	1,210,479	4	10,500,000	1961–2019	FAOSTAT
Sorghum Production (ton)	1522	245,611.4	506,910.9	0	5,265,580	1961–2019	FAOSTAT
Millet Production (ton)	1323	131,864.3	262,178.4	54	1,878,527	1961–2019	FAOSTAT
Rice Production (ton)	1669	372,630.1	938,768.1	0	7,253,373	1961–2019	FAOSTAT
Wheat Production (ton)	1105	607,994	1,521,939	0	9,607,736	1961–2019	FAOSTAT
Barley Production (ton)	552	449,506.7	749,312.6	100	3,831,130	1961–2019	FAOSTAT
Fonio Production (ton)	381	36,381.73	83,020.09	100	530,227	1961–2020	FAOSTAT
Poverty gap at the urban poverty line (%)	76	11.90395	9.049087	1.8	40	1961–2011	WDI
Poverty gap at the rural poverty line (%)	77	22.07273	9.499953	3.6	53	1961–2012	WDI
Battle-related deaths (number of people)	278	1411.522	4618.351	0	50,293	1961–2013	WDI

**Table 2:** Economic Growth Impacts by Climate-related Disasters and Aid.

Dependent Variable	GDP Per Capita Growth (Annual %)			
	(1)	(2)	(3)	(4)
Lagged GDP per capita growth	0.1694751 ***	0.1543027 **	0.16712 ***	0.1519377 **
	(2.80)	(2.49)	(2.75)	(2.44)
Education (+15 years old)	−1.228877	−1.139392	−1.25669	−1.172042
	(−0.56)	(−0.52)	(−0.58)	(−0.53)
Fertility Rate	1.35599	2.05509	1.479018	2.180705
	(0.66)	(0.97)	(0.71)	(1.02)
HDI	−3.463914	−0.953797	−2.788904	−0.2467038
	(−0.17)	(−0.04)	(−0.13)	(−0.01)
Number of People Affected by Climate-related Disasters	−8.057152 **	−8.057298 **		
	(−2.07)	(−2.05)		
Number of People Affected by Drought			−7.632749 *	−7.614573 *
			(−1.87)	(−1.85)
Number of People Affected by Flood			−13.04042	−13.28393
			(−0.99)	(−1.01)
Number of People Affected by Storm			0.00000684	0.00000685
			(1.05)	(1.05)
Government Expenditure	0.1529741	0.1360603	0.1624341	0.1460737
	(1.53)	(1.31)	(1.62)	(1.40)
Humanitarian ODA	−0.0000000007		−0.000000001	
	(−0.14)		(−0.14)	
Emergency ODA		−0.000000001		−0.000000001
		(−0.180)		(−0.18)
Constant	1.043053	−3.935066	0.1118494	−4.888921
	(0.06)	(−0.22)	(0.01)	(−0.28)
*N*	272	264	272	264
Type of Regression	FE	FE	FE	FE

Note: Numbers in parentheses are *t*-values; ***, **, and * indicate statistical significance at the 1%, 5%, and 10% levels, respectively.

**Table 3:** Impact on Agricultural Production by Climate-related Disasters and Aid.

Dependent Variable	Agriculture Production Index			
	(1)	(2)	(3)	(4)
GDP per capita growth	−0.0003642	−0.1086933	−0.115295	−0.032736
	(−0.00)	(−0.95)	(−0.99)	(−0.10)
Number of People Affected by Climate-related Disasters	−24.41415 ***	−23.02236 ***	−21.84345 **	−35.02979 **
	(−2.74)	(−2.58)	(−2.41)	(−2.06)
Educational Inequality, Gini	−4.608486 ***	−4.224185 ***	−4.253704 ***	−6.260741 ***
	(−19.84)	(−15.48)	(−15.29)	(−6.89)
Humanitarian ODA	−0.0000000052			
	(−0.47)			
ODA for Agriculture		0.000000075 ***	0.000000076 ***	
		(2.65)	(2.66)	
ODA for disaster prevention & preparedness (lagged)				−0.0000008
				(−0.95)
Local Government Index			−8.470382	3.579998
			(−1.35)	(0.14)
Constant	319.1347 ***	297.3906 ***	303.5181 ***	397.2466 ***
	(29.24)	(22.74)	(21.33)	(9.78)
*N*	370	379	370	120
Type of Regression	FE	FE	FE	FE

Note: Numbers in parentheses are *t*-values; ***, and ** indicate statistical significance at the 1%, and 5% levels, respectively.

**Table 4:** Impact on Agricultural Production by Climate-related Disasters and Aid.

Dependent Variable	Agriculture Production Index		
	(1)	(2)	(3)
GDP per capita growth	−0.0106388	−0.1173564	0.3344108 ***
	(−0.09)	(−1.01)	(5.03)
Number of People Affected by Climate-related Disasters	−23.4604 ***		
	(−2.58)		
Number of People Affected by Flood		−17.48007	−9.036425
		(−0.41)	(−0.30)
Number of People Affected by Drought		−24.51023 ***	−12.51029 *
		(−2.61)	(−1.94)
Number of People Affected by Storm		−0.000009	−0.000007
		(−0.93)	(−1.57)
Educational Inequality, Gini	−4.64529 ***	−4.230962 ***	−2.568219 ***
	(−19.48)	(−15.09)	(−26.68)
Local Government Index	−8.113644	−8.025702	20.9569 ***
	(−1.30)	(−1.28)	(6.93)
Emergency ODA	−0.000000003		
	(−0.775)		
ODA for Agriculture		0.00000008 ***	
		(2.70)	
Cereal Food Aid			−0.0000148 ***
			(−3.49)
Constant	326.9576 ***	302.2468 ***	206.6968 ***
	(26.54)	(21.07)	(37.24)
*N*	355	370	958
Type of Regression	FE	FE	FE

Note: Numbers in parentheses are *t*-values; *** and * indicate statistical significance at the 1%, and 10% levels, respectively.

**Table 5:** Impact on Cereal Production by Climate-related Disasters and Aid.

Dependent Variable	(1)	(2)	(3)	(4)	(5)	(6)	(7)	(8)
	Cereal Production Index	Maize Production	Sorghum Production	Millet Production	Rice Production	Wheat Production	Barley Production	Fonio Production
GDP per capita growth	0.3519966	1591.614	−1304.271	830.9917	−1334.704	9952.268	24,650.14	−2459.17
	(1.22)	[0.41]	(−0.74)	(0.62)	[−0.45]	[1.06]	[0.80]	(−1.21)
Educational Inequality, Gini	−7.099192 ***	−32,610.23 ***	−5121.155	−989.7303	−8121.831	−8631.816	−1084.82	−12,828.91 ***
	(−10.44)	[−5.19]	(−1.58)	(−0.40)	[−1.37]	[−0.54]	[−0.05]	(−8.28)
Number of People Affected by Flood	−4.82892	−910,600.5	27,443.77	−35,073.24	−223,888.4	1,576,617	−1,673,180	−745,509.2 ***
	(−0.05])	[−0.46]	(0.03)	(−0.06)	[−0.16]	[0.19]	[−0.07]	(−2.92)
Number of People Affected by Drought	−51.96536 **	−1,027,551 ***	−39,551.82	−102,533.6	−46,956.45	91,261.4	−10,5139.6	118,431.8
	(−2.27)	[−3.75]	(−0.33)	(−0.94)	[−0.19]	[0.15]	[−0.09]	(0.46)
Number of People Affected by Storm	0.000000779	−0.1388403	−0.0418895	−0.1643853	−0.66323 ***	−0.0056365	0.0449533	−51.86224 *
	(0.03)	[−0.64]	(−0.44)	(−0.62)	[−2.77]	[−0.01]	[0.01]	(−1.92)
ODA for Agriculture	0.000000116 *	0.0040394 ***	0.001325 ***	0.000796 ***	0.0034716 ***	0.0011092	0.0014411	−0.0003032
	(1.65)	[5.45]	(3.95)	(3.19)	[5.11]	[0.53]	[0.42]	(−2.39)
Constant	433.7546 ***	2,230,139 ***	461,934.9 ***	208,877.2	866,423.4 **	1,128,003	400,714.1	1,043,952 ***
	(13.19)	[5.79]	(2.82)	(1.55)	[2.43]	[1.32]	[0.36]	(8.78)
*N*	373	247	324	185	332	158	62	45
Type of Regression	FE	FE	RE	FE	RE	RE	RE	FE

Note: Numbers in brackets are *z*-values, and in parentheses are *t*-values; ***, **, and * indicate statistical significance at the 1%, 5%, and 10% levels, respectively.

**Table 6:** Cereal Food Aid and Cereal Production.

Dependent Variable	(1)	(2)	(3)	(4)	(5)	(6)	(7)	(8)
	Cereal Production Index	Maize Production	Sorghum Production	Millet Production	Rice Production	Wheat Production	Barley Production	Fonio Production
GDP per capita growth	0.9851934 ***	6623.565 ***	2369.321	1372.234	−645.8084	15,459.38 ***	42,489.02 ***	−775.6227
	(4.12)	[2.74]	(2.18)	(1.62)	[−0.42]	[2.75]	[4.30]	(−0.64)
Educational Inequality, Gini	−1.605586 ***	−20,892.1 ***	−4520.574 ***	−4523.103 ***	−11,114.77 ***	−10,901.36 **	10845.18	−6237.34 ***
	(−4.97)	[−10.16]	(−4.98)	(−5.85)	[−4.99]	[−2.16]	[1.36]	(−7.90)
Number of People Affected by Flood	54.60008	−55925.41	−5490.607	−1908.055	−518772.2	−1131083	3,794,894	−313,625.6
	(0.50)	[−0.07]	(−0.02)	(−0.01)	[−0.64]	[−0.57]	[0.32]	(−1.02)
Number of People Affected by Drought	−31.99213	−552,229.1 ***	−61,476.59	−74,580.37	−1235.48	48,256.81	64,481.36	86,418.55
	(−1.39)	[−3.75]	(−1.04)	(−1.21)	[−0.01]	[0.15]	[0.13]	(0.77)
Number of People Affected by Storm	−0.000008	−0.119833	−0.0115932	0.000002	0.0225578	0.0035995	−5.263566	−66.31653
	(−0.50)	[−1.37]	(−0.33)	(0.00)	[0.25]	[0.02]	[−0.90]	(−1.54)
Cereal Food Aid	−0.000033 ***	−0.94005 ***	−0.073275 **	0.1050056 *	−1.45745 ***	−1.6366 ***	−0.0561999	0.1900641
	(−2.14)	[−10.41]	(−2.00)	(1.74)	[−15.80]	[−8.37]	[−0.30]	(0.71)
Constant	177.215 ***	1,782,212 ***	458,177.8 ***	403,646.2 ***	1,150,479 ***	1,287,005 ***	−120,938.3	535,114.8 ***
	(10.77)	[10.82]	(9.45)	(9.17)	[5.83]	[3.85]	[−0.25]	(8.68)
*N*	952	631	540	457	598	401	165	115
Type of Regression	FE	RE	FE	FE	RE	RE	RE	FE

Note: Numbers in brackets are *z*-values, and in parentheses are *t*-values; ***, **, and * indicate statistical significance at the 1%, 5%, and 10% levels, respectively.

**Table 7:** Poverty and Battle.

Dependent Variable	Poverty in Rural Area	Poverty in Urban Area	Poverty in Urban Area	Battle Related Deaths	Battle Related Deaths
	(1)	(2)	(3)	(4)	(5)
GDP per capita growth	−0.5020925 *	−0.0326405	−0.1644819	−87.08594 **	−87.32139 **
	[−1.84]	[−0.15]	(−0.63)	(−2.37)	(−2.40)
Number of People Affected by Climate-related Disasters	9.285749	15.6348 **		11,854.74 ***	
	[1.12]	[2.34]		(2.93)	
Number of People Affected by Flood			−64.6435		12,956.34
			(−1.50)		(0.57)
Number of People Affected by Drought			20.16717 **		13,122.96 ***
			(2.57)		(3.89)
Number of People Affected by Storm			−79.01091		−0.0149493
			(−0.88)		(−0.28)
Constant	23.394 ***	11.288 ***	12.342 ***	1142.126 ***	1105.389 ***
	[16.03]	[8.01]	(12.38)	(3.91)	(3.77)
*N*	77	76	75	260	260
Type of Regression	RE	RE	FE	FE	FE

Note: Numbers in parentheses are *t*-values; ***, **, and * indicate statistical significance at the 1%, 5%, and 10% levels, respectively.

**Table 8:** Factors Contributed to Reduce the Number of Deaths by Disasters.

Dependent Variable	Total Number of Deaths by Climate-Related Disasters			
	(1)	(2)	(3)	(4)
HDI (Human Development Index)	−504.342	−621.4799	−349.3779 *	−354.2997 *
	(−1.31)	(−1.50)	(−1.68)	(−1.68)
Gov. Effectiveness	−131.8227 **	−141.7638 **	−54.9951 *	−55.76964 *
	(−2.37)	(−2.48)	(−1.77)	(−1.76)
Control of Corruption	36.64476	20.4265	31.70747	31.43229
	(0.60)	(0.32)	(0.96)	(0.93)
Regulatory Quality		49.01479	14.14797	13.79879
		(0.76)	(0.48)	(0.46)
ODA for Disaster Prevention and Preparedness	−0.000008 ***	−0.0000073 ***		
	(−2.78)	(−2.65)		
Humanitarian ODA			−0.0000002 ***	
			(−4.04)	
Emergency ODA				−0.0000002 ***
				(−4.16)
Constant	187.7321	254.3052	181.7999 *	181.3178 *
	(1.05)	(1.28)	(1.74)	(1.73)
*N*	234	234	343	334
Type of Regression	FE	FE	FE	FE

Note: Numbers in brackets are *z*-values, and in parentheses are *t*-values; ***, **, and * indicate statistical significance at the 1%, 5%, and 10% levels, respectively.

## Data Availability

The data that support the study findings are available from Go Shimada. Restrictions apply to the availability of these data, as they are used under license for this study.

## Appendix A

**Table A1:** Non-Cereal Crops.

Dependent Variable	(1)	(2)	(3)	(4)
	Banana Production	Cassava Production	Tea Production	Coffee Production
GDP per capita growth	−3964.216 *	−13,413.36	493.2195	87.8662
	(−1.87)	[−1.51]	[0.98]	[0.26]
Educational Inequality, Gini	−35,370.73 ***	−123,926.1 ***	−1405.38	623.709
	(−7.14)	[−5.87]	[−1.61]	[1.15]
Number of People Affected by Flood	−172,524.4	4,010,153	−157,908.5	53,130.28
	(−0.14)	[0.69]	[−0.60]	[0.26]
Number of People Affected by Drought	117,739.9	−1,717,949	33,912.43	−81,737.55 **
	(0.59)	[−1.59]	[1.46]	[−2.08]
Number of People Affected by Storm	0.0329793	−1.00887	−0.0004288	0.0114696
	(0.25)	[−1.63]	[−0.04]	[0.55]
ODA for Agriculture	0.0002185	0.0047701 **	0.0001	−0.0000291
	(0.45)	[2.15]	[1.54]	[−0.35]
Constant	2,052,232 ***	9,049,522 ***	106,277.7 **	2775.313
	(8.55)	[6.25]	[1.89]	[0.10]
*N*	197	188	80	144
Type of Regression	FE	RE	RE	FE

Note: Numbers in brackets are *z*-values, and in parentheses are *t*-values; ***, **, and * indicate statistical significance at the 1%, 5%, and 10% levels, respectively.
